# CD8^+^ Regulatory T Cells Induced by Lipopolysaccharide Improve Mouse Endotoxin Shock

**DOI:** 10.4049/immunohorizons.2200074

**Published:** 2023-05-22

**Authors:** Nanaka Morita, Masato Hoshi, Hiroyuki Tezuka, Tatsuya Ando, Sayaka Yoshida, Fumiaki Sato, Hiroyuki Yokoi, Hiroyasu Ito, Kuniaki Saito

**Affiliations:** * Department of Disease Control and Prevention, Fujita Health University, Toyoake, Aichi, Japan; † Department of Informative Clinical Medicine, Fujita Health University, Toyoake, Aichi, Japan; ‡ Cellular Function Analysis, Research Promotion Headquarters, Fujita Health University, Toyoake, Aichi, Japan; § Joint Research Laboratory of Clinical Medicine, Fujita Health University, Toyoake, Aichi, Japan

## Abstract

Sepsis is a systemic inflammatory disease caused by a bacterial infection that leads to severe mortality, especially in elderly patients, because of an excessive immune response and impaired regulatory functions. Antibiotic treatment is widely accepted as the first-line therapy for sepsis; however, its excessive use has led to the emergence of multidrug-resistant bacteria in patients with sepsis. Therefore, immunotherapy may be effective in treating sepsis. Although CD8^+^ regulatory T cells (Tregs) are known to have immunomodulatory effects in various inflammatory diseases, their role during sepsis remains unclear. In this study, we investigated the role of CD8^+^ Tregs in an LPS-induced endotoxic shock model in young (8–12 wk old) and aged (18–20 mo old) mice. The adoptive transfer of CD8^+^ Tregs into LPS-treated young mice improved the survival rate of LPS-induced endotoxic shock. Moreover, the number of CD8^+^ Tregs in LPS-treated young mice increased through the induction of IL-15 produced by CD11c^+^ cells. In contrast, LPS-treated aged mice showed a reduced induction of CD8^+^ Tregs owing to the limited production of IL-15. Furthermore, CD8^+^ Tregs induced by treatment with the rIL-15/IL-15Rα complex prevented LPS-induced body wight loss and tissue injury in aged mice. In this study, to our knowledge, the induction of CD8^+^ Tregs as novel immunotherapy or adjuvant therapy for endotoxic shock might reduce the uncontrolled immune response and ultimately improve the outcomes of endotoxic shock.

## Introduction

In bacterial infections, appropriate immune responses are induced in the host to eliminate invading microorganisms and maintain immunological homeostasis. However, excessive immune response to microorganisms often leads to severe inflammatory diseases and tissue injury. Sepsis is a systemic inflammatory disease caused by bacterial infections. It is characterized by elevated levels of inflammatory cytokines, such as IL-6, IL-1β, and TNF-α, in the serum (1), leading to multiple-organ failure and death (2). Notably, elderly patients and aged mice with severe sepsis have high mortality rates because of an uncontrolled immune response through the overproduction of inflammatory cytokines (3–6). Despite much research on the pathogenesis of sepsis, this syndrome remains a leading cause of death in intensive care units (7).

LPS, a component of the outer membrane of gram-negative bacteria, causes a robust immune response (8). During the early phase of a bacterial infection, LPS stimulates neutrophils, macrophages, and dendritic cells (DCs) to produce large amounts of inflammatory cytokines via TLR4 (2). LPS-stimulated DCs promote the differentiation of naive CD4^+^ T cells into pathogenic effector T cells through excessive production of inflammatory cytokines (9). A cytokine storm induced by aberrant activation of innate and adaptive immune cells in response to LPS leads to endotoxic shock (10). In the early phase of bacterial infections, the percentage of CD4^+^ regulatory T cells (Tregs) is increased (11), and adoptive transfer of CD4^+^ Tregs improves the survival rate (12). These reports suggest that CD4^+^ Tregs have a role in improving sepsis.

Recently, CD8^+^CD122 (IL-2Rβ-chain)^+^ cells have been identified as a new subset of Tregs with immunosuppressive functions, which are mediated by the production of IL-10 (13–16). In mammals, CD8^+^ Tregs have been reported to be associated with viral infections (17, 18), autoimmune diseases (13, 19–25), cancer (26, 27), and graft-versus-host disease (28–31). Accumulating evidence suggests that in addition to CD4^+^ Tregs, CD8^+^ Tregs play a critical role in immunomodulatory effects in various inflammatory diseases. However, the biological significance of CD8^+^ Tregs in sepsis remains unknown.

In this study, we found that the frequency of CD8^+^ Tregs in both peripheral blood and spleen increased during the late phase of LPS-induced endotoxic shock. An adoptive transfer experiment showed that IL-10–producing CD8^+^ Tregs were essential for host survival.

## Materials and Methods

### Mice

Young (8–12 wk old) male and female mice and aged (18–20 mo old) male mice were used in this study. C57BL/6N mice were obtained from Japan Charles River (Kanagawa, Japan). The mice were housed in a specific pathogen-free environment in our facility, maintained at 23°C ± 2°C on a 12-h light/dark cycle (lights on at 8:00 am), and had free access to food and water. All experiments were performed in accordance with the Guidelines for Animal Care of Fujita Health University (Approval No. AP17018). The Animal Experimentation Committee of Fujita Health University approved the protocol for all animal experiments. Procedures involving mice and their care conformed to international guidelines, as described in the *Principles of Laboratory Animal Care* (National Institutes of Health publication 85-23, revised 1985).

### Mouse endotoxic model

To induce mild endotoxic shock, we injected young male and female mice i.p. with 10 mg/kg LPS (dissolved in PBS, single administration, *E. coli* O55:B5; Sigma-Aldrich, St. Louis, MO), except where noted. In one experiment, young mice were injected i.p. with 20 mg/kg LPS to induce severe endotoxic shock. Control mice were administered an identical amount of PBS. Furthermore, in the aged mouse experiments, we injected 1 mg/kg LPS because the LD_50_ of LPS in aged mice has been reported to be 1.6 to 1.84 mg/kg (4, 5).

To obtain samples, we anesthetized and euthanized animals at the indicated times. The time of LPS administration was defined as 0 h in subsequent experiments.

### Cell preparation and flow cytometry

Blood, diluted in EDTA, and the spleen were harvested from mice at various time points. Isolated splenocytes were prepared by removing the spleen membrane and passing through a 70-μm filter. Splenocytes and blood cells were washed, and RBCs were lysed as previously described (32). Single cells were incubated with anti-CD16/32 (93; BioLegend, San Diego, CA) to block Fc receptors to prevent nonspecific Ab binding and then were stained with the following mAbs conjugated to FITC, PE, allophycocyanin, or PE-indotricarbocyanine: anti-mouse CD3 (17A2; BioLegend), anti-mouse CD8 (53-6.7; BioLegend), anti-mouse CD122 (TM-β1; BioLegend), anti-mouse CD4 (GK1.5; BioLegend), anti-mouse CD25 (PC61; BioLegend), and anti-mouse CD49b (HMa2; BioLegend) Abs. For intranuclear staining, the cells were fixed and permeabilized with Foxp3 Fix/Perm solution (BioLegend) and then stained with PE-conjugated anti-mouse Helios Ab (22F6; BioLegend). The cells were analyzed using FACSCalibur in conjunction with FlowJo analysis software (BD Bioscience, Tokyo, Japan) or Gallios in conjunction with Kaluza analysis software (Beckman Coulter, Brea, CA).

### Cell culture reagents and conditions

The cells were cultured in RPMI 1640 medium (Wako Pure Chemical Industries, Osaka, Japan) supplemented with 10% heat-inactivated FBS (Boehringer Mannheim Biochemica, Manheim, Germany), 100 U/ml penicillin (Sigma-Aldrich), and 100 mg/ml streptomycin (Sigma-Aldrich). AIM-V serum-free medium (Life Technologies, Paisley, U.K.) was used to differentiate into CD8^+^ Tregs. All cells were cultured at 37°C with 5% CO_2_.

### Isolation and adoptive transfer of stimulated CD8^+^ Tregs

Splenocytes were obtained from young mice on day 7 after administration of 10 mg/kg LPS and labeled with CD8 and CD122 fluorochrome-conjugated Abs. CD8^+^ Tregs (identified as CD8^+^CD122^+^ cells) were isolated using a MoFlo Astrios high-speed cell sorter (Beckman Coulter) to a purity >98%. CD8^+^ Tregs were then stimulated and adoptively transferred as previously described (12, 33). CD8^+^ Tregs (3 × 10^5^) were stimulated with recombinant mouse IL-15 (100 ng/ml; PeproTech, Cranbury, NJ) and anti-CD3/CD28–coated microbeads (bead-to-cell ratio of 1:1; Life Technologies) in 24-well plates for 72 h at 37°C with 5% CO_2_. After culture, CD8^+^ Tregs were collected and counted using trypan blue solution (Wako Pure Chemical Industries), and CD8^+^ Tregs (3 × 10^5^) were transferred i.v. into young mice 6 h after 20 mg/kg LPS administration to induce severe endotoxic shock. Survival rates were recorded for 7 d and analyzed. Furthermore, in the aged mouse experiments, CD8^+^ Tregs (1.75 × 10^5^) from LPS-treated young mice were transferred i.v. into aged mice 6 h after 1 mg/kg LPS administration to induce endotoxic shock. Survival rates and body weight were recorded for 7 consecutive days and analyzed. To obtain samples, we anesthetized and euthanized animals at the indicated times. The time of LPS administration was defined as 0 h in subsequent experiments.

In an experiment comparing CD8^+^ Tregs from young and aged mice, splenocytes were harvested from septic young and aged mice on day 7 after 1 mg/kg LPS administration and stained according to the earlier protocol.

### Histological analysis

Livers, lungs, and kidneys were fixed in 10% formalin in PBS for 24 h at 24°C and then embedded in paraffin. Sections (liver and kidney: 3 μm thickness, lung: 1 μm thickness) were stained with H&E.

### Isolation of CD11c^+^, CD11c^−^, CD11b^+^, and CD11b^−^ cells

Splenic CD11c^+^, CD11c^−^, CD11b^+^, and CD11b^−^ cells from mice at 6 h after 10 mg/kg LPS administration were isolated using MACS Magnetic Bead columns (Miltenyi Biotec, Bergisch Gladbach, Germany) according to the manufacturer’s instructions.

### In vitro LPS stimulation of CD11c^+^ cells and detection of IL-15

CD11c^+^ and CD11c^−^ cells were stimulated with LPS as previously described (34). In brief, splenic CD11c^+^ and CD11c^−^ cells from untreated young mice were isolated using MACS Magnetic Beads columns (Miltenyi Biotec) according to the manufacturer’s instructions and seeded at a density of 1 × 10^6^ cells/well in a 96-well plate. Splenic CD11c^+^ and CD11c^−^ cells were stimulated with LPS (100 ng/ml) for 24 h at 37°C with 5% CO_2_. After culturing, culture supernatants were collected. Media supernatants were stored at −80°C until analysis. The supernatants in the absence of cells were used as a negative control sample. IL-15 cytokine release with a mouse IL-15 ELISA kit was used to analyze the supernatant (Invitrogen, Thermo Fisher Scientific, Waltham, MA).

### Isolation and stimulation of CD4^+^ and CD8^+^ Tregs

Splenocytes were obtained from young septic mice on day 7 after administration of 10 mg/kg LPS and labeled with CD4, CD25, CD8, and CD122 fluorochrome-conjugated Abs. CD4^+^ Tregs (identified as CD4^+^CD25^+^ cells) or CD8^+^ Tregs were isolated to a purity >98% using a MoFlo Astrios high-speed cell sorter (Beckman Coulter). CD4^+^ and CD8^+^ Tregs were stimulated as previously described (35, 36). In brief, CD4^+^ or CD8^+^ Tregs (3 × 10^5^) were stimulated with PMA (50 ng/ml; Sigma-Aldrich) and ionomycin (500 ng/ml; Sigma-Aldrich) in 96-well round-bottom plates for 72 h at 37°C with 5% CO_2_. After culturing, CD4^+^ Tregs, CD8^+^ Tregs, and media supernatants were collected. Media supernatants were stored at −80°C until analysis.

### RNA extraction and real-time PCR analysis

Total RNA was extracted from the spleen, which was obtained from LPS-untreated or -treated mice at 0, 6, and 24 h using Isogen II (Nippon Gene, Tokyo, Japan) or CD11c^+^ cells, CD11c^−^ cells, CD11b^+^, CD11b^−^ cells, CD4^+^ Tregs, and CD8^+^ Tregs using the FastGene RNA Basic kit (Nippon Genetics, Tokyo, Japan) according to the manufacturer’s instructions, and the RNA concentration was determined spectrophotometrically at 260 nm. RT-PCR was performed using a high-capacity cDNA reverse transcription kit (Applied Biosystems, Foster City, CA). The following PCR primers were used: IL-15 sense, 5′-CAGTTGCAGAGTTGGACGAAG-3′ and antisense, 5′-GACCATGAAGAGGCAGTGCT-3′; IL-10 sense, 5′-TGCACTACCAAAGCCACAAG-3′ and antisense, 5′-TAAGAGCAGGCAGCATAGCAG-3′; 18S rRNA sense, 5′-TATCGGAATTAACCAGACAA-3′ and antisense, 5′-TATCGGAATTAACCAGACAA-3′. The mRNA expression levels of IL-15, IL-10, and 18S rRNA were quantified by quantitative PCR using a 7900HT Fast Real-Tome system (Applied Biosystems). PCR was performed using the SsoAdvanced Universal SYBR Green Supermix (Bio-Rad Laboratories, Hercules, CA). Data were analyzed using 7900HT software (version 2.3; Applied Biosystems).

### CD8^+^ naive T cell differentiation into CD8^+^ Tregs

CD8^+^ Tregs were differentiated as previously described (37). According to the manufacturer’s instructions, CD8^+^CD44^−^ naive T cells were isolated using MACS Magnetic Bead columns (Miltenyi Biotec). CD8^+^ naive T cells (5 × 10^5^ cells/well) were stimulated with anti-CD3/CD28–coated beads (bead-to-cell ratio of 1:5; Life Technologies) ± IL-15 (100 ng/ml; PeproTech) in AIM-V serum-free medium (Life Technologies) for 5 d in 48-well plates at 37°C with 5% CO_2_. After culturing, the stimulated cells were harvested and stained according to the protocol described earlier.

### Measurement of IL-10

Culture medium supernatants of CD4^+^ or CD8^+^ Tregs stimulated with PMA and ionomycin were obtained according to the earlier protocol (see *Isolation and stimulation of CD4*^+^ *and CD8*^+^ *Tregs*). Media supernatants in the absence of cells were used as a negative control sample. IL-10 cytokine release with a mouse IL-10 ELISA kit was used to analyze the supernatant (eBioscience).

### In vitro LPS stimulation of CD11c^+^ cells from young and aged mice

CD11c^+^ cells were stimulated with LPS as previously described (34). In brief, splenic CD11c^+^ cells from untreated young and aged mice were isolated using MACS Magnetic Beads columns (Miltenyi Biotec) according to the manufacturer’s instructions and seeded at a density of 1 × 10^5^ cells/well in a 96-well plate. Splenic CD11c^+^ cells were stimulated with LPS (100 ng/ml) for 6 h at 37°C with 5% CO_2_. Control CD11c^+^ cells were untreated. At the end of the stimulation, cells were collected and analyzed for IL-15 mRNA levels by quantitative PCR.

### Assessment of cell proliferation of CD8^+^ Tregs by water-soluble tetrazolium salt-1

CD8^+^ Tregs from LPS-treated young and aged mice were seeded at a density of 1 × 10^4^ cells/well in a 96-well plate in the presence of IL-15 (100 ng/ml; PeproTech) and anti-CD3/CD28 beads (bead-to-cell ratio of 1:2; Life Technologies). After 24 h at 37°C with 5% CO_2_, 10 µl of water-soluble tetrazolium salt-1 (WST-1) reagent (Premix WST-1 Cell Proliferation Assay System; Takara Bio, Shiga, Japan) was added, and the cells were incubated for 4 h at 37°C with 5% CO_2_. The absorbance was measured using a microplate reader (main wavelength, 450 nm; auxiliary wavelength, 620 nm).

### Preparation and treatment with IL-15/IL-15Rα complex

Recombinant mouse IL-15 was purchased from BioLegend (catalog no. 566302). Mouse IL-15Rα subunit Fc chimera (IL-15 Rα) was purchased from R&D Systems (catalog no. 551-MR-100; Minneapolis, MN). Recent work has shown that the effects of IL-15 can be markedly enhanced by combining IL-15 with its IL-15Rα subunit (38, 39). The IL-15/IL-15Rα complex was prepared as previously described (34). In brief, 20 μg IL-15 and 200 μg IL-15Rα-Fc were incubated in 430 μl of PBS at 37°C with 5% CO_2_ for 20 min to IL-15/IL-15Rα complex. Samples were diluted 10-fold in PBS to a total volume of 4.3 ml, then aliquoted and frozen. For induction of CD8^+^ Tregs, aged mice were treated s.c. with 300 μl of IL-15/IL-15Rα complex solution (1 μg IL-15 and 7.0 μg IL-15Rα-Fc) at 6, 24, and 48 h after 1 mg/kg LPS administration. To obtain samples, we anesthetized and euthanized animals at the indicated times. The time of LPS administration was defined as 0 h in subsequent experiments. Survival rates and body weight were recorded for 7 consecutive days and analyzed.

### Statistical analysis

GraphPad Prism software (version 6.0; GraphPad, San Diego, CA) was used for data analysis. All data are expressed as means ± SD. Statistical differences between the two groups were determined using Student *t* test or a two-sided paired *t* test. Multiple groups were compared with one-way ANOVA or two-way ANOVA, followed by Tukey’s multiple comparison test. The survival rates of mice were analyzed by the Kaplan–Meier method. A *p* value <0.05 was considered statistically significant.

## Results

### LPS-induced CD8^+^ Tregs improve the mortality of mice with endotoxic shock

**FIGURE 1.**
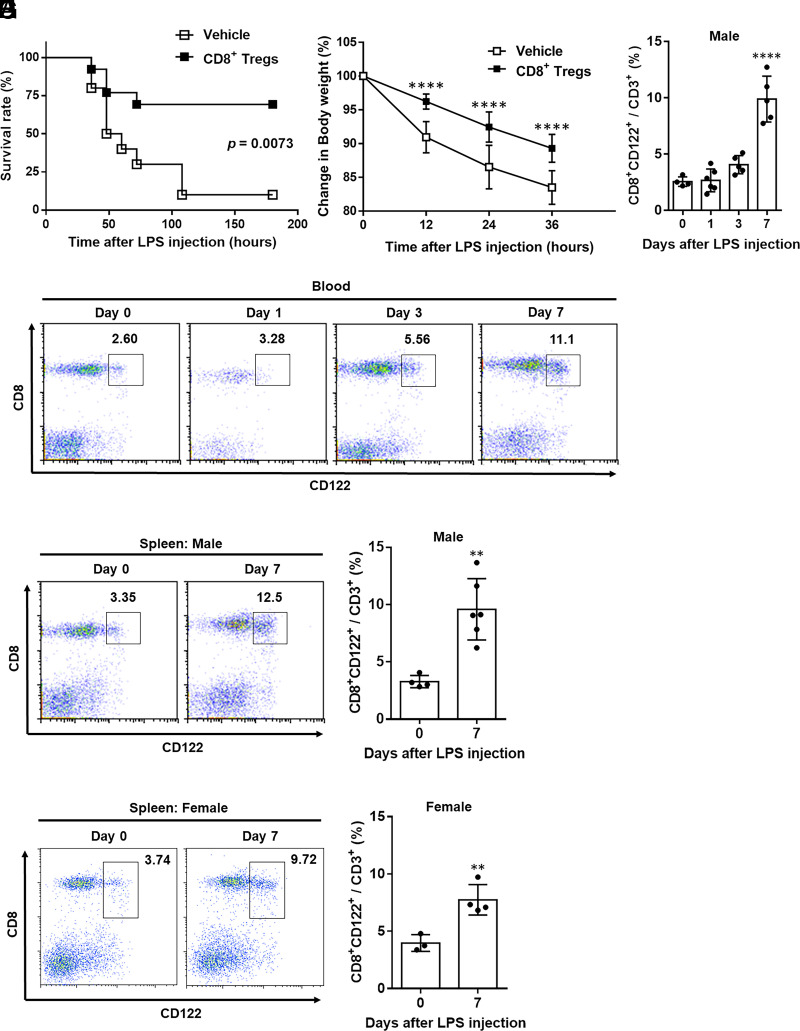
LPS-induced CD8^+^ Tregs improve the survival rate of endotoxic shock in mice. (**A** and **B**) Young mice were injected with LPS (20 mg/kg, i.p.), and stimulated CD8^+^ Tregs (3 × 10^5^ cells, i.v., *n* = 13) or PBS (*n* = 10) were transferred i.v. into LPS-treated young mice at 6 h after LPS administration. (A) A statistically significant difference between the groups was determined using the log-rank test. (B) Change in body weight of vehicle-treated mice (*n* = 10) or CD8^+^ Treg-transferred mice (*n* = 13) was compared at indicated time after LPS administration. ****p < 0.0001, using two-way ANOVA, followed by Tukey multiple comparisons. (**C**–**H**) CD8^+^ Tregs, defined as CD8^+^CD122^+^ cells, were analyzed using flow cytometry on blood (C and D) and splenocytes (E–H) from LPS-treated or untreated young male (C–F) or female (G and H) mice at indicated days, respectively, gated on 7-Amino-Actinomycin D^−^CD3^+^ cells. Representative dot plots (C, E, and G) and cumulative data graphical summaries (D, F, and H) are depicted. Data (*n* = 3 − 6 per group) were presented as mean ± SD. Statically significant differences between groups were determined using one-way ANOVA, followed by Tukey multiple comparisons test (D) and Student *t* test (F and H). **p < 0.01, ****p < 0.0001.

To confirm the effect of CD8^+^ Tregs on endotoxic shock, we first performed an adoptive transfer of ex vivo–expanded CD8^+^CD122^+^ cells into septic mice. In brief, CD8^+^CD122^+^ cells from LPS-treated mice were expanded ex vivo and then transferred into mice that received a high dose of LPS. Interestingly, the survival rates of LPS-induced endotoxin shock were drastically improved by the adoptive transfer of CD8^+^CD122^+^ cells (Fig. 1A). Furthermore, LPS-induced body weight loss and tissue injury were prevented by the adoptive transfer of CD8^+^CD122^+^ cells (Fig. 1B, Supplemental Fig. 1).

Next, we examined the frequency of CD4^+^ and CD8^+^ T cells in the LPS-induced septic mice. After LPS administration, the frequency of CD4^+^ T cells in the blood and spleen gradually decreased, whereas the frequency of CD8^+^ T cells increased in a time-dependent manner (Supplemental Fig. 2A–D). Notably, the proportion of CD8^+^CD122^+^ cells in the blood and spleen significantly increased at day 7 after LPS administration in male and female mice (Fig. 1C–H). To confirm whether increase of the frequency CD8^+^CD122^+^ cells is influenced by loss of other T cell subsets, we investigated the absolute number of CD8^+^CD122^+^ cells. Importantly, absolute CD8^+^CD122^+^ cell counts were also increased at day 7 after LPS administration (Supplemental Fig. 2E). Furthermore, flow cytometric analysis showed that most CD8^+^CD122^+^ cells did not express CD49b, a marker of NK T cells (Supplemental Fig. 3), indicating that they are likely CD8^+^ Tregs. These results suggest that CD8^+^CD122^+^ Tregs are essential for controlling endotoxic shock.

### IL-15 produced by LPS-stimulated CD11c^+^ cells proliferate CD8^+^ Tregs

**FIGURE 2.**
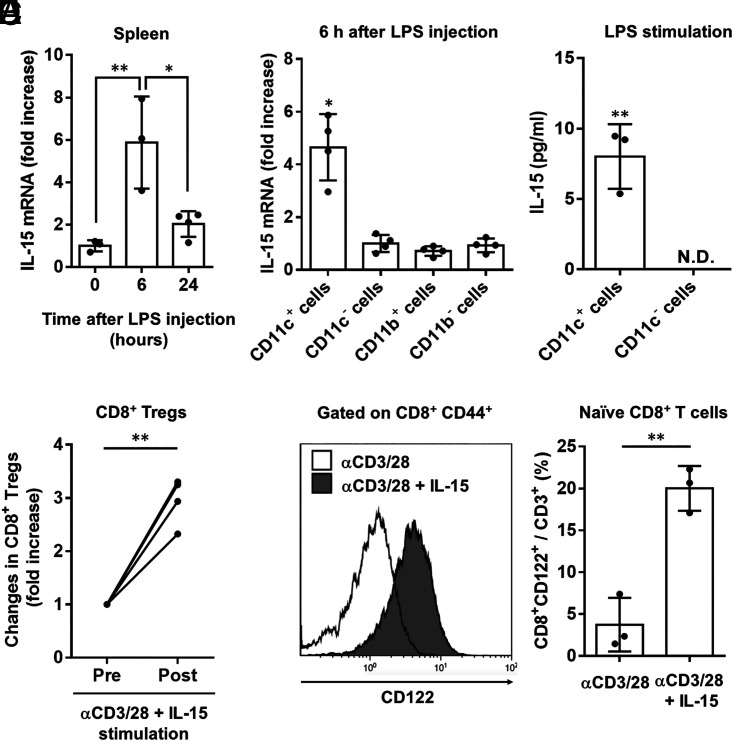
IL-15 produced by LPS-stimulated CD11c^+^ cells induce CD8^+^ Tregs. (**A** and **B**) The expression levels of IL-15 mRNA in the total spleen (*n* = 3–4 per group) and various isolated cells (*n* = 4 per group) at indicated time after LPS administration were determined using quantitative RT-PCR. (**C**) Splenic CD11c^+^ or CD11c^−^ cells were stimulated with LPS (100 ng/ml) for 24 h. IL-15 levels in the culture supernatants were detected using ELISA (*n* = 3 independent experiments). (**D**) Changes in the number of CD8^+^ Tregs prestimulation or poststimulation of IL-15 (100 ng/ml) with anti-CD3/CD28–coated beads (*n* = 4 independent experiments). (**E** and **F**) Naive CD8^+^CD44^−^ T cells were stimulated with anti-CD3/CD28–coated beads in the presence or absence of IL-15 (100 ng/ml) for 5 d (*n* = 3 per group). The frequency of CD8^+^ Tregs was determined by FACS analysis. Representative histogram plots (E) and cumulative data graphical summary (F) are depicted. Data were presented as mean ± SD. Statically significant differences between the groups were determined using one-way ANOVA, followed by Tukey multiple comparisons test (A and B), and determined using a two-sided paired *t* test (D) or Student *t* test (C and F). *p < 0.05, **p < 0.01.

To investigate the mechanism by which LPS induces CD8^+^ Tregs, we focused on IL-15, which selectively promotes their differentiation into CD8^+^ T cells (33). IL-15 mRNA levels in the spleen significantly increased 6 h after LPS administration (Fig. 2A). To identify the source of IL-15 in the spleen during endotoxic shock, we assessed CD11c^+^ DCs and CD11b^+^ macrophages, both of which are known to produce IL-15 in inflammatory conditions (40, 41). IL-15 expression levels were the highest in CD11c^+^ cells (Fig. 2B), and IL-15 protein levels were also significantly higher in CD11c^+^ cells than in CD11c^−^ cells (Fig. 2C), suggesting that DCs are the primary producers of IL-15 during endotoxic shock.

Next, we examined the effects of IL-15 on CD8^+^ Treg proliferation. CD8^+^ Tregs isolated from LPS-treated mice were cultured in the presence of IL-15, which significantly increased the number of CD8^+^ Tregs (Fig. 2D). Naive CD8^+^ T cells obtained from naive mice were cultured in the presence or absence of IL-15. The differentiation of CD8^+^ naive T cells into CD8^+^ Tregs was significantly increased in the presence of IL-15 (Fig. 2E, 2F).

### LPS-induced CD8^+^ Tregs are increased in the spleen and produce IL-10, comparable with CD4^+^ Tregs

**FIGURE 3.**
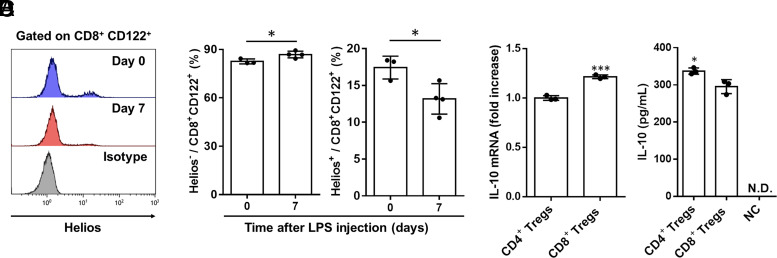
LPS-induced CD8^+^ Tregs are increased in the spleen and produce IL-10. (**A** and **B**) Helios expression on CD8^+^ Tregs was analyzed using flow cytometry on splenocytes from LPS-treated (*n* = 4) or untreated mice (*n* = 3) at indicated days, respectively. Representative histogram plots (A) and cumulative data graphical summary (B) are depicted. (**C**) IL-10 mRNA levels in the CD4^+^ Tregs (identified as CD4^+^CD25^+^ cells) or CD8^+^ Tregs (identified as CD8^+^CD122^+^ cells) from LPS-treated mice by stimulation of PMA (50 ng/ml) and ionomycin (500 ng/ml) for 72 h were determined using quantitative RT-PCR (*n* = 3 independent experiments). (**D**) The IL-10 protein levels in the culture media of CD4^+^ or CD8^+^ Tregs from LPS-treated mice stimulated with PMA (50 ng/ml) and ionomycin (500 ng/ml) for 72 h were measured using ELISA. Culture media in the absence of cells were used as negative control (*n* = 3 independent experiments). Data were presented as mean ± SD. *p < 0.05, ***p < 0.001, using Student *t* test. NC, negative control; N.D., not detected.

To elucidate the origin of CD8^+^ Tregs during endotoxic shock, we examined the expression of transcription factor Helios, a marker of thymus-derived Tregs, on CD8^+^ Tregs (42). More than 85% of LPS-induced CD8^+^ Tregs did not express Helios (Fig. 3A, 3B), suggesting that CD8^+^ Tregs were induced in the spleen, which is presumably a subset of peripherally derived Tregs.

Helios^−^CD4^+^ Tregs prevent inflammatory responses by producing IL-10 (43). To evaluate the IL-10–producing ability of Helios^−^CD8^+^ Tregs induced by LPS administration, we cultured CD4^+^ and CD8^+^ Tregs in the presence of PMA and ionomycin. IL-10 mRNA and protein levels were comparable between CD4^+^ and CD8^+^ Tregs in septic mice (Fig. 3C, 3D).

### Aged mice have limited induction of CD8^+^ Tregs by LPS stimulation

**FIGURE 4.**
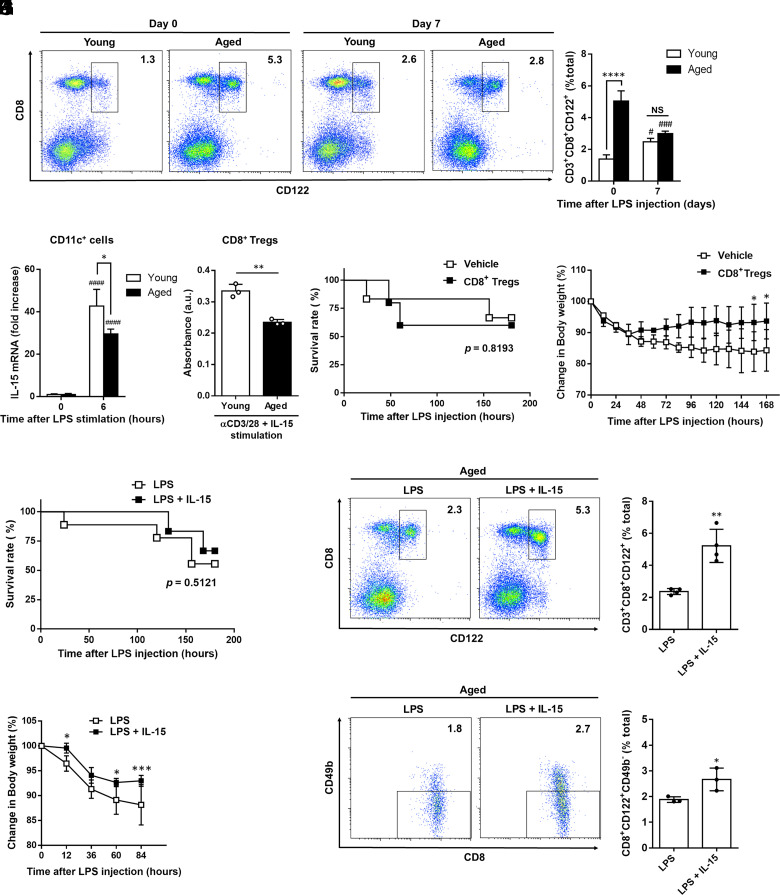
Treatment with IL-15/IL-15Rα complex improved limited induction of CD8^+^ Tregs in aged mice. (**A** and **B**) CD8^+^ Tregs were analyzed using flow cytometry on splenocytes from young or aged mice at indicated days after LPS administration, respectively, gated on 7-Amino-Actinomycin D^−^CD3^+^ cells. Representative dot plots (A) and cumulative data graphical summary (B) are depicted. Data (*n* = 3–4 per group) were presented as mean ± SD. ****p < 0.0001 compared with young mice; ^#^*p* < 0.05, ^###^*p* < 0.001 compared with day 0, using one-way ANOVA, followed by Tukey multiple comparisons test. (**C**) The expression level of IL-15 mRNA in the splenic CD11c^+^ cells from young or aged mice at indicated time after LPS stimulation (100 ng/ml) was determined using quantitative RT-PCR. Data (*n* = 3 independent experiments) were presented as mean ± SD. *p < 0.05 compared with young mice; ^####^*p* < 0.0001 compared with 0 h, using one-way ANOVA, followed by Tukey multiple comparisons test. (**D**) The proliferation of CD8^+^ Tregs from young or aged mice by stimulating with anti-CD3/CD28–coated beads in the presence of IL-15 (100 ng/ml) for 24 h was determined using WST-1 assay. Data (*n* = 3 independent experiments) were presented as mean ± SD. **p < 0.01, using Student *t* test. (**E** and **F**) Aged mice were injected with LPS (1 mg/kg, i.p.), and stimulated CD8^+^ Tregs (1.75 × 10^5^ cells, i.v.; *n* = 7) from LPS-treated young mice or PBS (*n* = 6) were transferred into aged mice at 6 h after LPS administration. (E) The survival rates of vehicle-treated or CD8^+^ Treg-transferred mice were compared at indicated time after LPS administration. A statistically significant difference between the groups was determined using the log-rank test. (F) Change in body weight of vehicle-treated or CD8^+^ Treg-treated mice was compared at indicated time after LPS administration. *p < 0.05, using two-way ANOVA, followed by Tukey multiple comparisons test. (**G**–**L**) Aged mice were treated s.c. with IL-15/IL-15Rα complex solution (1 μg IL-15 and 7.0 μg IL-15Rα-Fc) or vehicle at 6, 24, and 48 h after 1 mg/kg LPS administration. The survival rate (G) and change in body weight (L) of LPS plus IL-15–treated (LPS + IL-15) or LPS alone–treated (LPS) aged mice at indicated time after LPS administration. A statistically significant difference between the groups was determined using the log-rank test (G). *p < 0.05, ***p < 0.001, using two-way ANOVA, followed by Tukey multiple comparisons test (L). The frequency of CD8^+^ Tregs (H and I) and CD49b expression on CD8^+^CD122^+^ cells (J and K) in the spleen from indicated mice (day 7) were analyzed using flow cytometry. Representative dot plots (H and J) and cumulative data graphical summary (I and K) are depicted. Data (*n* = 3 per group) were presented as mean ± SD. *p < 0.05, **p < 0.01, using Student *t* test.

Older patients are highly susceptible to sepsis (44). Based on our results, we hypothesized that in elderly patients, reduced numbers of CD8^+^ Tregs are responsible for the high susceptibility to endotoxic shock. Therefore, we analyzed the frequency of CD8^+^ Tregs after LPS administration in aged mice. Consistent with previous reports (13, 45), untreated aged mice had substantially more splenic CD8^+^ Tregs than young untreated mice. However, on LPS administration, the frequency of CD8^+^ Tregs was comparable between the aged and young mice (Fig. 4A, 4B). These results indicate that the LPS-driven induction of CD8^+^ Tregs is impaired in aged mice. IL-10 mRNA levels in LPS-induced CD8^+^ Tregs were not significantly different between the young and aged mice (Supplemental Fig. 4A). In addition, on LPS administration, the percentage of Helios^−^CD8^+^ Tregs increased in young mice (Fig. 3A, 3B), whereas Helios^−^CD8^+^ Tregs were not induced in aged mice (Supplemental Fig. 4B, 4C).

To investigate whether the impaired induction of CD8^+^ Tregs in aged mice was due to reduced IL-15 production, we cultured CD11c^+^ cells from untreated young and aged mice in the presence of LPS. We found that the LPS-induced expression of IL-15 mRNA in CD11c^+^ cells was significantly lower in aged mice than in young mice (Fig. 4C). To further investigate whether the responsiveness of CD8^+^ Tregs to IL-15 was reduced in aged mice, we cultured CD8^+^ Tregs sorted from LPS-treated young and aged mice with anti-CD3/CD28 and IL-15. The cell proliferation of CD8^+^ Tregs in aged mice was significantly reduced compared with that in young mice (Fig. 4D). Taken together, these results suggest that impaired induction of CD8^+^ Tregs in aged mice is due to not only reduced IL-15 production from CD11c^+^ cells but also reduced cell proliferation of CD8^+^ Tregs in response to IL-15.

### Treatment with IL-15/IL-15Rα complex improves the induction of CD8^+^ Tregs in aged mice

To assess the impact of impaired induction of CD8^+^ Tregs and IL-15 production in aged mice on endotoxin shock, we first performed the adoptive transfer experiment of CD8^+^ Tregs from LPS-treated young mice to LPS-treated aged mice. The survival rates of LPS-treated aged mice were not improved by the transfer of CD8^+^ Tregs (Fig. 4E). However, LPS-induced body weight loss was significantly prevented by injection of exogenous CD8^+^ Tregs (Fig. 4F).

Next, we examined the effects of IL-15 treatment on the induction of CD8^+^ Tregs. In brief, aged mice were treated with or without IL-15/IL-15Rα complex solution after LPS administration. Consistent with a previous report using young mice (46), spleen size was larger than in LPS plus IL-15/IL-15Rα complex–treated aged mice compared with LPS alone–treated aged mice (Supplemental Fig. 4D). Interestingly, treatment with IL-15/IL-15Rα complex did not improve the survival rate of endotoxin shock (Fig. 4G), whereas it improved the induction of CD8^+^ Tregs (Fig. 4H–K) and prevented weight loss and tissue injury (Fig. 4L, Supplemental Fig. 4E).

## Discussion

In this study, CD8^+^ Tregs in young mice increased through the production of IL-15 by stimulation with LPS and improved the survival rate of LPS-induced endotoxin shock. In contrast, in aged mice, the induction of CD8^+^ Tregs was impaired because of reduced IL-15 signaling. Furthermore, CD8^+^ Tregs induced by treatment with rIL-15/IL-15Rα complex prevented LPS-induced body wight loss and tissue injury in aged mice. To our knowledge, these results indicate that CD8^+^ Tregs may be a target for novel therapeutic strategies for endotoxin shock.

Previous studies have reported that adoptive transfer of CD8^+^ Tregs in Experimental Autoimmune Encephalomyelitis model mice reduces symptoms by suppressing the production of IFN-γ and IL-17 through the production of IL-10 (19, 25). The immunosuppressive effects of IL-10–producing CD8^+^ Tregs have been reported in various inflammatory diseases (13, 16, 19, 25). IL-10, which is produced by various cell types, including CD4^+^ Tregs, DCs, B cells, and macrophages, is essential for regulating excessive immune responses to numerous pathogens (47–49). Among these cells, CD4^+^ Treg–derived IL-10 is critical in regulating excessive immune responses against pathogens (50). In this context, our findings showed that the capacity of CD8^+^ Tregs to produce IL-10 was comparable with that of CD4^+^ Tregs. Furthermore, we showed that the adoptive transfer of CD8^+^ Tregs improved the survival rate of LPS-induced endotoxin shock, suggesting that IL-10–producing CD8^+^ Tregs are also important for the regulation of excessive immune responses during endotoxic shock. However, the functional differences between CD8^+^ and CD4^+^ Tregs in the improvement of endotoxic shock could not be fully clarified in this study. In this regard, TLR4^+^CD4^+^ Tregs induced within day 3 after cecal ligation puncture (CLP) are directly stimulated by LPS (51, 52). In contrast, TLR-4^−^CD8^+^ Tregs are indirectly activated by LPS (53). Consistent with these results, we found that the frequency of CD8^+^, but not CD4^+^ (data not shown), Tregs increased in the late and/or recovery phase during endotoxin shock. These findings suggest that CD4^+^ and CD8^+^ Tregs are induced to prevent excessive immune responses during the early and late phases of endotoxin shock, respectively.

Elderly and aged mice are highly susceptible to bacterial infections and exhibit high mortality rates (3, 4, 44). Moreover, cytokine storm is thought to cause high mortality rates in elderly models of sepsis (5, 6). Therefore, controlling cytokine storms could be used to reduce mortality rates. In this context, we found that LPS-induced CD8^+^ Tregs depended on the production of IL-15 by CD11c^+^ cells. Although the expression of TLR-4 in splenic CD11c^+^ cells is comparable between young and aged mice (32), we found that LPS-induced expression of IL-15 mRNA in CD11c^+^ cells was lower in aged mice than in young mice. Furthermore, treatment with rIL-15/IL-15Rα complex improved the CD8^+^ Treg induction, body weight loss, and tissue injury in aged mice. In a previous study using young mice, treatment with rIL-15/IL-15Rα complex improves the survival rate in a mouse CLP model (54), supporting our results. In contrast, Guo et al. (55) reported that treatment with rIL-15/IL-15Rα complex exacerbates the survival rate in a mouse CLP model. The differences observed between these studies are not entirely clear because of the differences in mouse age, dosage, or delivery routes of rIL-15/IL-15Rα complex and models of sepsis. Regardless of these differences, our study indicates that the induction of CD8^+^ Tregs, which contribute to the control of the cytokine storm, in aged mice may be limited by reduced IL-15 production. A recent human clinical trial with IL-15 agonists has shown that, among T cell subsets, the expansion of CD8^+^ T cells, but not CD4^+^ Tregs, was induced selectively (56). However, in the clinical trial, CD8^+^ Tregs have not been investigated (56). Although the immune responses in mice and humans do not necessarily coincide, we found that administration of IL-15 induced CD8^+^ Tregs in aged mice. Based on these results, although it was speculated that CD8^+^, but not CD4^+^, Tregs are preferentially induced by stimulation with IL-15 agonists, further studies are required to clarify.

Our study had an inherent limitation. The LPS-induced endotoxic model is not an equivalent of sepsis, and another model, i.e., CLP, could not have been evaluated in this study. Although the adoptive transfer of CD8^+^ Tregs into septic mice drastically improved their survival rates, it remains unclear whether their production of IL-10 mediates this effect. We could not demonstrate how transferred CD8^+^ Tregs regulate other immune cell functions. However, we found that LPS-induced CD8^+^ Tregs produced IL-10, which directly suppresses inflammatory cytokines production via downregulation of MHC class II and costimulatory molecule B7-1/B7-2 on macrophages and DCs, and indirectly suppresses T cells and NK cells (47, 57). Therefore, it seems that CD8^+^ Tregs have inhibitory effects on other immune cells via their production of IL-10. It has been reported that macrophages and DCs that have received a low dose of LPS fail to produce inflammatory cytokines and mediators in response to a subsequent high dose of LPS (as it is called “priming”) (58, 59). However, in this study, young mice adaptively transferred with CD8^+^ Tregs were administered a single dose of LPS. Therefore, we believe that mechanisms by which transferred CD8^+^ Tregs improve survival rates of young mice are independent of LPS priming, and further investigations are needed to fully clarify this.

In this study, we showed that IL-15 produced by LPS-stimulated CD11c^+^ cells induced CD8^+^ Tregs, and that LPS-induced CD8^+^ Tregs may ameliorate endotoxin shock. Furthermore, impaired induction of CD8^+^ Tregs in aged mice may be one of the reasons for their high mortality. Thus, although further investigation is needed, CD8^+^ Tregs may become a new target for preventing endotoxic shock.

## Supplementary Material

Supplemental 1 (PDF)
