# Supplementary material for: CD8+ Regulatory T Cells Induced by Lipopolysaccharide Improve Mouse Endotoxin Shock
Source: Immunohorizons. 2023 May 22;7(5):353–63. doi: 10.4049/immunohorizons.2200074 (PMC10579971; doi:10.4049/immunohorizons.2200074)
Supplement: Supplemental 1 (PDF) [file IH_2200074_Supplemental_1.pdf]

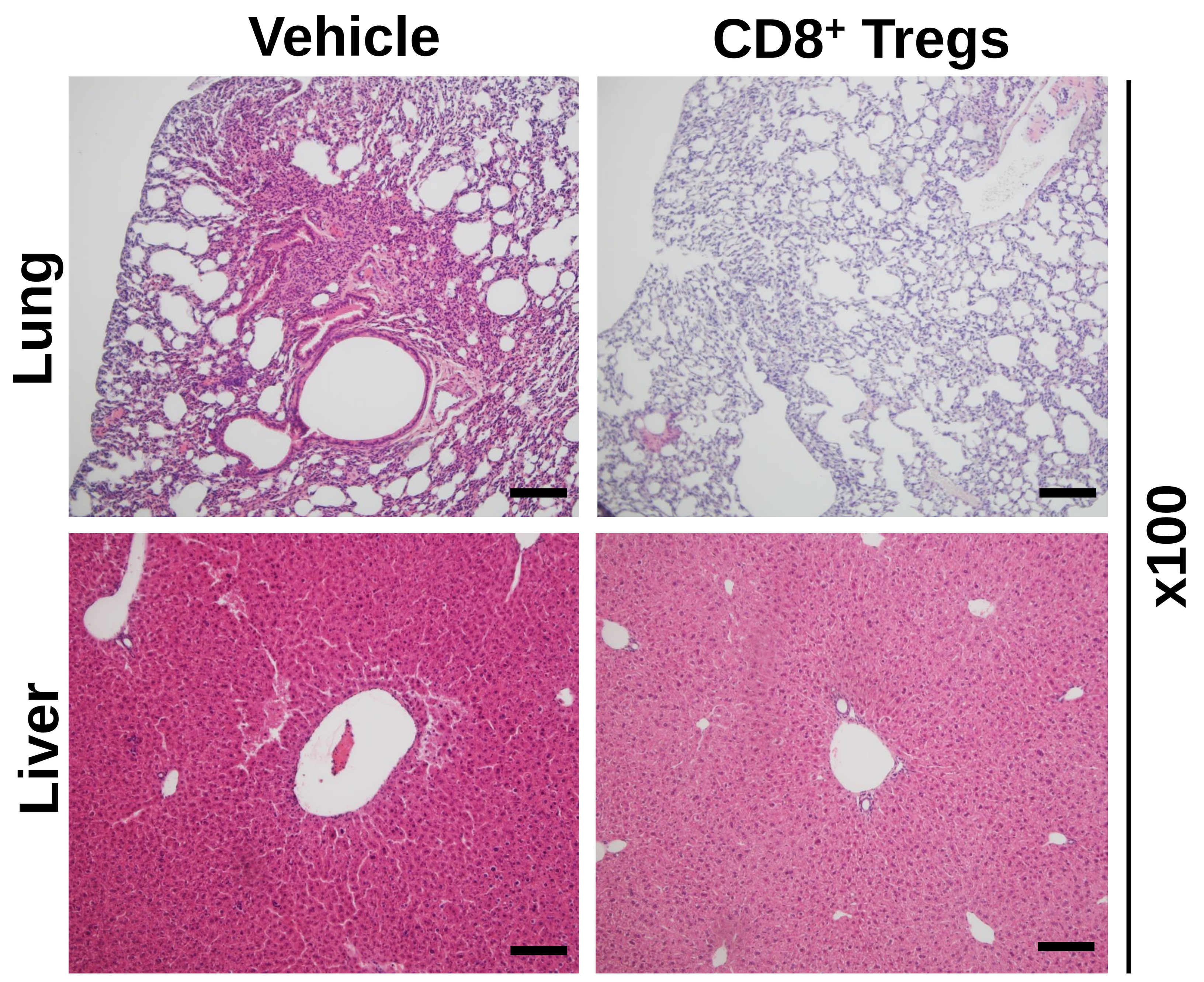

Supplemental Figure. 1 The tissue injury was protected by after adoptive transfer of $CD8^+$ $CD122^+$ cells. Histological change in the lung (upper panels) and liver (lower panels) of vehicle-treated mice or $CD8^+$ $CD122^+$ cells-transferred mice were compared at 7 days after LPS administration. Scale bar, 100 μm.

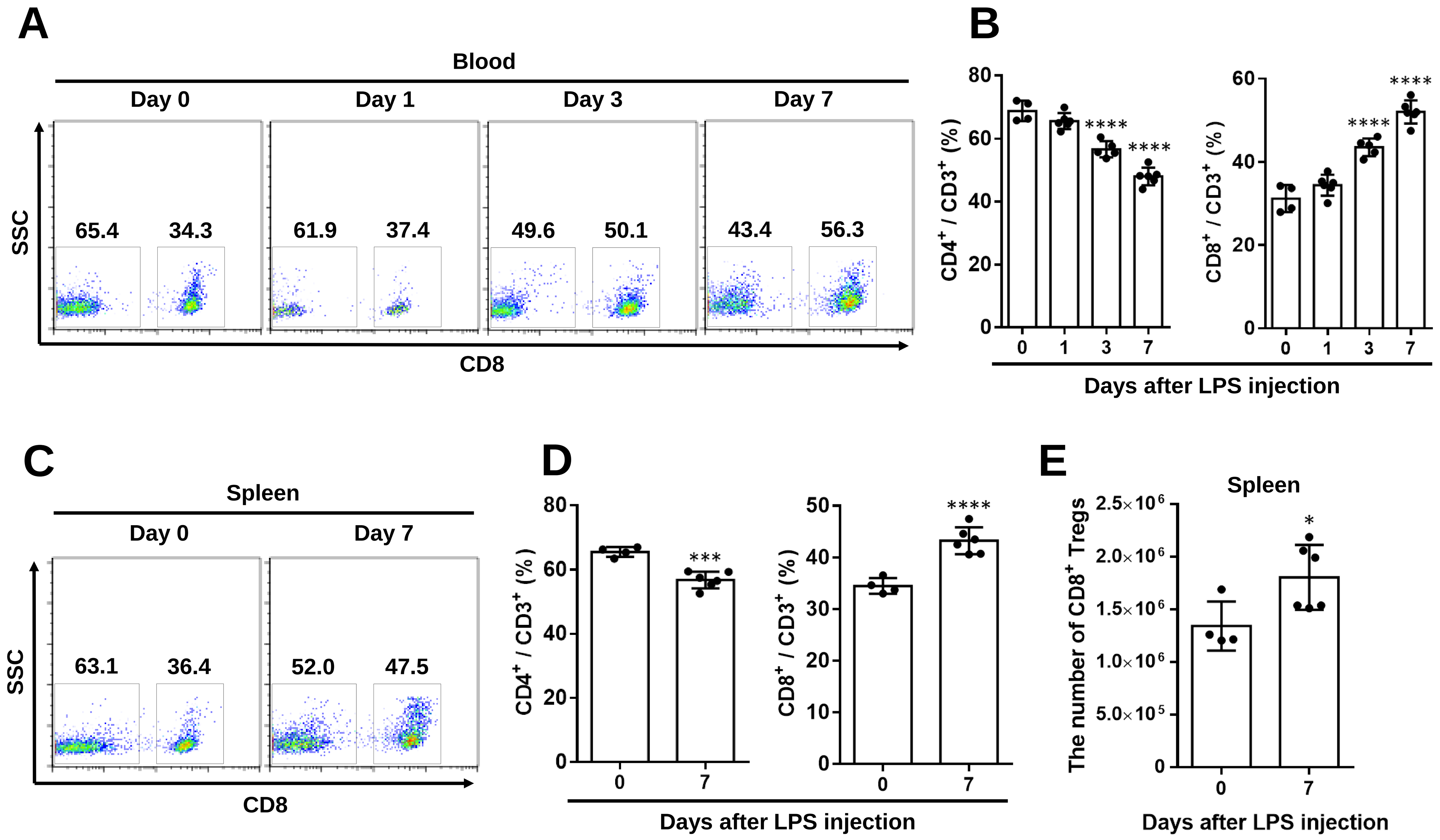

Supplemental Figure. 2 Change in T cell subsets after LPS administration.
(A-D) $CD4^+$ and $CD8^+$ T cells were analyzed using flow cytometry on the blood (A and B) and spleen (C and D) from LPS-treated or untreated young male mice on the indicated days, respectively, gated on 7-AAD^- $CD3^+$ cells. Representative dot plots (A and C) and cumulative data summaries (B and D) are shown. Data (n = 4-6 per group) are presented as mean ± SD. Statistically significant differences between the groups were determined using one-way ANOVA, followed by Tukey's multiple comparisons test (B), and determined using Student's t-test (D). ***p < 0.001, **** p < 0.0001. (E) The number of $CD8^+$ Tregs per the spleen from LPS-treated or untreated young male mice on the indicated days. Date (n = 4-6 per group) were presented as mean ± SD. * p < 0.05, using Student t-test.

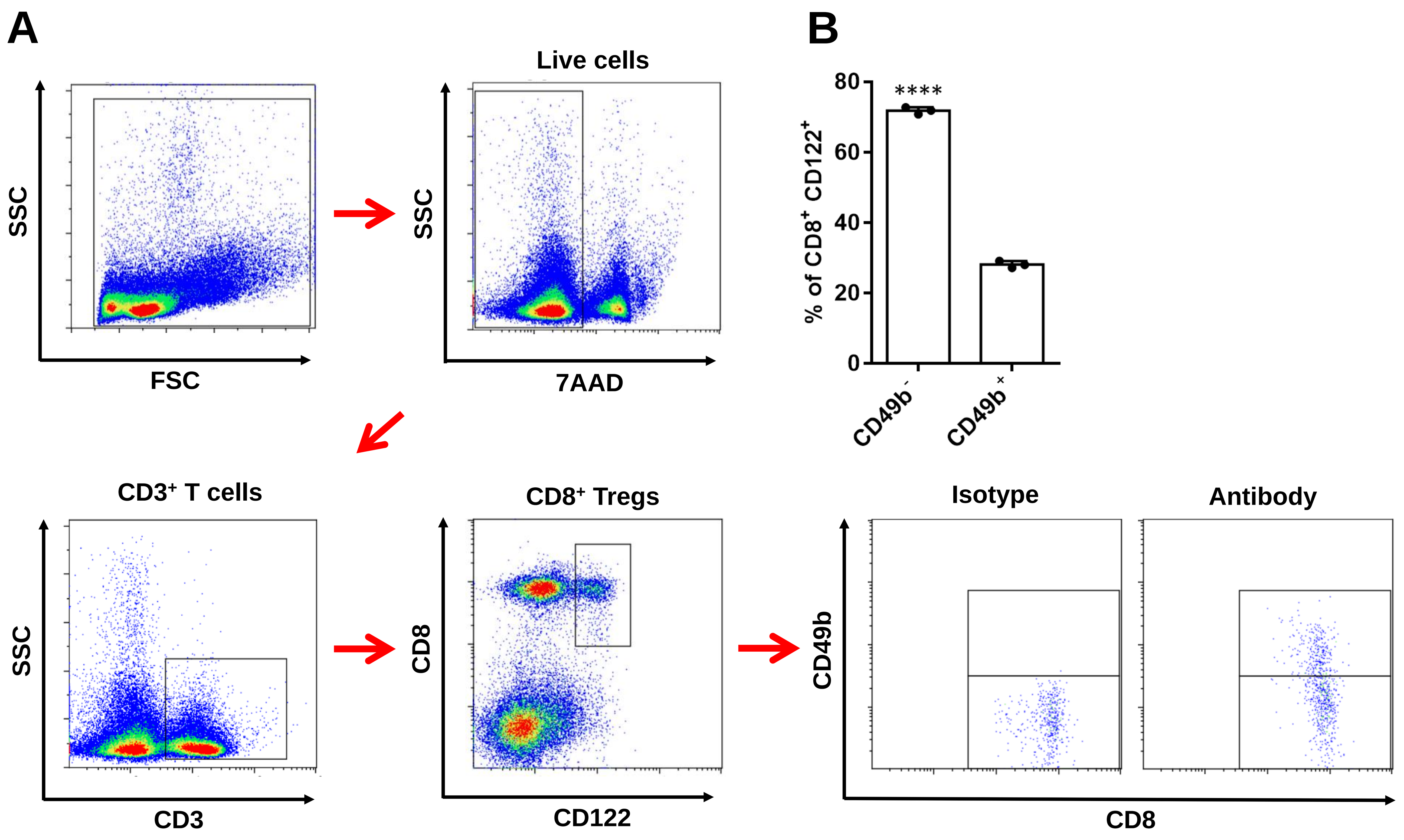

Supplemental Figure. 3 The expression of CD49b in LPS-induced CD8^{+} CD122^{+} cells. (A and B) CD49b expression in CD8^{+} CD122^{+} cells was analyzed using flow cytometry on splenocytes from young mice (n = 3) 7 days after LPS administration. Representative flow cytometry plots (A) and a cumulative data summary (B) are shown.

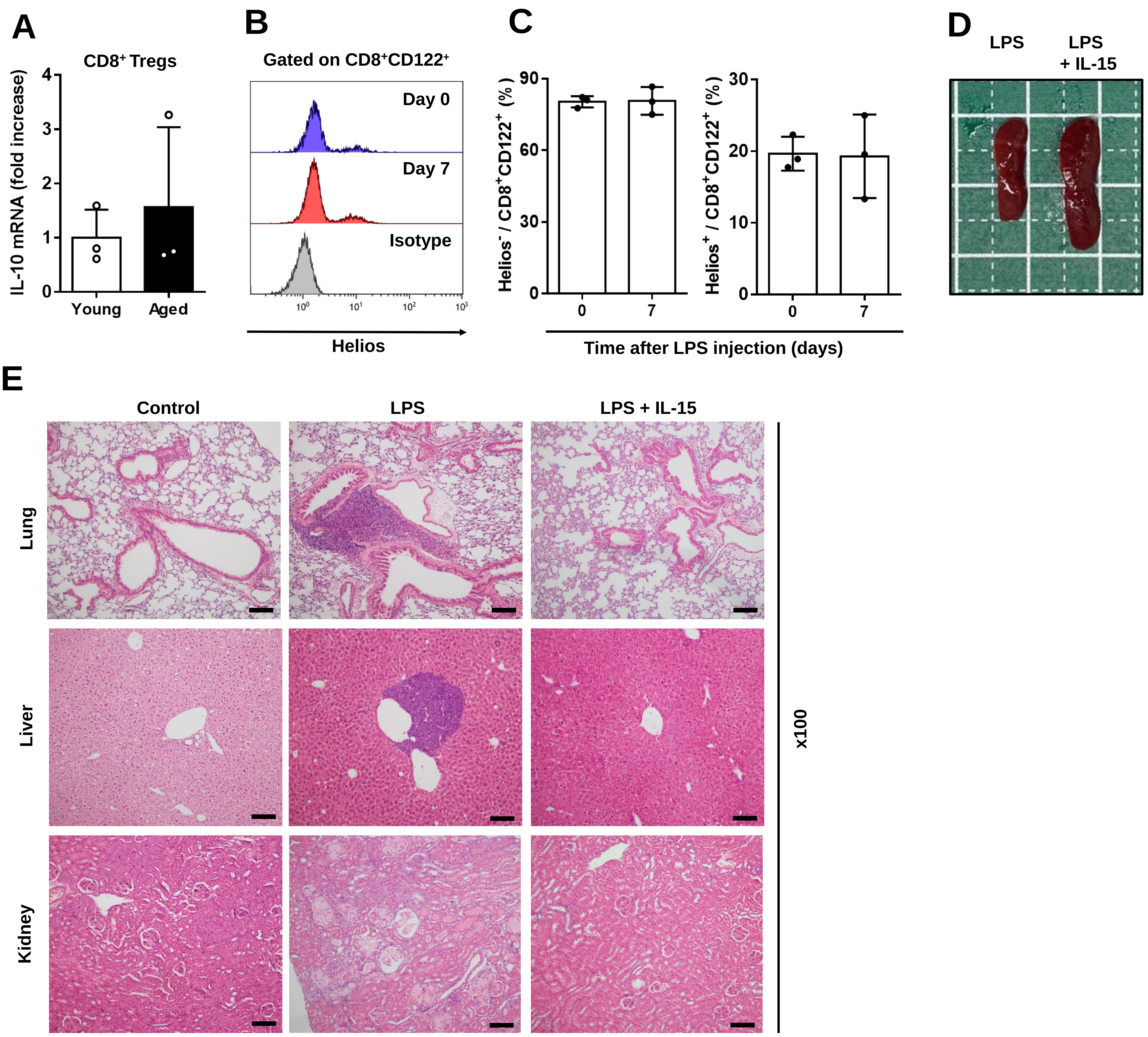

Supplemental Figure. 4 The function of CD8^{+} Tregs in aged mice after LPS administration.
(A) The expression of IL-10 levels in CD8^{+} Tregs from LPS-treated young (n = 3) and aged mice (n = 3) were determined by quantitative RT-PCR. (B and C) Helios expression on CD8^{+} Tregs was analyzed using flow cytometry on splenocytes from LPS-treated (n = 3) or untreated aged mice (n = 3) on the indicated days. Representative histogram plots of aged mice (B) and cumulative data summary (C) are shown. (D and E) Aged mice were treated s.c. with IL-15/IL-15Rα complex solution (1 μg IL-15 and 7.0 μg IL-15Rα-Fc) or vehicle at 6 h, 24 h, and 48 h after 1 mg/kg of LPS administration. (D) Representative image of the spleen at day 7. (E) Histological change in the lung (upper panels), liver (middle panels) and kidney (lower panels) of LPS alone-treated and LPS plus IL-15-treated mice were compared at day 7 day after LPS administration. Scale bar, 100 μm.
